# Folding of a LysM Domain: Entropy-Enthalpy Compensation in the Transition State of an Ideal Two-state Folder

**DOI:** 10.1016/j.jmb.2008.05.020

**Published:** 2008-07-11

**Authors:** Adrian A. Nickson, Kate E. Stoll, Jane Clarke

**Affiliations:** University of Cambridge Department of Chemistry, MRC Centre for Protein Engineering, Lensfield Road, Cambridge, CB2 1EW, UK

**Keywords:** CAM3, circular variant of LysM, GdmCl, guanidinium chloride, LysM, lysin motif domain, RCO, relative contact order, ACO, absolute contact order, LRO, long-range order, protein folding, phi-value, foldon, Eyring plot, entropy-enthalpy balance

## Abstract

Protein-engineering methods (Φ-values) were used to investigate the folding transition state of a lysin motif (LysM) domain from *Escherichia coli* membrane-bound lytic murein transglycosylase D. This domain consists of just 48 structured residues in a symmetrical βααβ arrangement and is the smallest αβ protein yet investigated using these methods. An extensive mutational analysis revealed a highly robust folding pathway with no detectable transition state plasticity, indicating that LysM is an example of an ideal two-state folder. The pattern of Φ-values denotes a highly polarised transition state, with significant formation of the helices but no structure within the β-sheet. Remarkably, this transition state remains polarised after circularisation of the domain, and exhibits an identical Φ-value pattern; however, the interactions within the transition state are uniformly weaker in the circular variant. This observation is supported by results from an Eyring analysis of the folding rates of the two proteins. We propose that the folding pathway of LysM is dominated by enthalpic rather than entropic considerations, and suggest that the lower entropy cost of formation of the circular transition state is balanced, to some extent, by the lower enthalpy of contacts within this structure.

## Introduction

Most small, single-domain proteins fold in a two-state manner^1^ as a consequence of their minimally frustrated energy landscapes.^2^ Evolution has selected for polypeptide sequences where native interactions are mutually supportive and lead cooperatively towards a single low-energy structure.^2–4^ Whilst this funnelling ensures a robust native state topology, it also provides a landscape where any energetic blockages can be circumvented by alternative folding routes. Indeed, it is now apparent that pathway malleability is a general phenomenon in protein folding, rather than the exception to the rule.^5,6^

Such malleability is usually identified through changes in the structure of the folding transition state, and can be described as movement either ”along” or ”across” the energy landscape.^5^ Transverse shifts across this landscape, i.e. parallel pathways, can be induced by point mutations,^7–9^ circular permutation (also termed entropic mutation),^10,11^ or changes in environmental conditions.^12,13^ Transverse shifts are also observed when using the fold approach^14^ to study proteins with similar topologies but divergent amino-acid sequences.^15,16^ This type of pathway malleability usually results in an alteration in the pattern of Φ-values. In contrast, movement along the energy landscape, i.e. Hammond behaviour,^17,18^ induced through a change in solvent conditions or mutation, is commonly manifested as downward curvature in the unfolding limb of a chevron plot.

Both classes of pathway malleability have been studied extensively in the αβ protein S6_T_: Hammond shifts were used to provide details of transition state movement along the pathway,^19^ whilst transverse shifts between pathways were investigated using so-called loop-entropy perturbations.^11^ A global analysis of these results provided a mechanistic connection between the two classes of malleability, and revealed the existence of at least two nucleation motifs within the S6_T_ structure.^5^ Both motifs comprise three topological elements: two β-strands and an α-helix. This two-strand-helix motif is not unique to S6_T_ and has been identified as the folding nucleus for several other protein domains.^5,20^ The authors use the term foldon to refer to these kinetically competent, quasi-independently folding units. However, in contrast to the original definition of a foldon, which specified a discrete contiguous section of polypeptide chain,^21^ the term was expanded to include any nucleation-competent submotif.

Oliveberg and co-workers surmise that it is the native state topology of a protein that determines the number and arrangement of the constituent foldons, and thus the response of the folding pathway to mutation.^22^ They propose that where a protein domain comprises a single foldon, the structure of the transition state is unaffected by amino acid substitutions and the protein can be classified as an ideal two-state folder.^5^ Such proteins are necessarily small (less than ≈ 80 residues) since, they suggest, a single foldon is unable to induce the cooperative folding of a more substantial polypeptide chain. The authors propose that larger proteins will either possess spatially separate foldons leading to multi-state folding (as seen in barnase^23^ and Im7^24^), or contain overlapping foldons that result in more complex two-state kinetics (as in U1A,^12^ ubiquitin^25^ and cytochrome c^26^).

To investigate these hypotheses, we studied the folding of a lysin motif (LysM) domain from *Escherichia coli* membrane-bound lytic murein transglycosylase D (MltD).^27^ This domain was chosen for two reasons. Firstly, LysM has a β_1_α_1_α_2_β_2_ topology and therefore the common two-strand-helix nucleation motif is likely to be entropically disfavoured. Secondly, the near-symmetry of the protein could theoretically provide two distinct transition state ensembles that are nearly degenerate in free-energy cost, leading to observable pathway malleability.^3^

At just 48 structured residues, LysM is currently the smallest αβ protein that has been investigated extensively using protein-engineering methods. In contrast to our expectations, the results indicate that LysM is an ideal two-state folder. We suggest that the only viable nucleation-competent submotif within the LysM structure is the α_1_α_2_β_2_ foldon, and that the folding pathway of this domain is dominated by enthalpic rather than entropic considerations.

## Results

### Studies on the wild-type LysM domain

#### Preliminary results

**Fig. 1:**
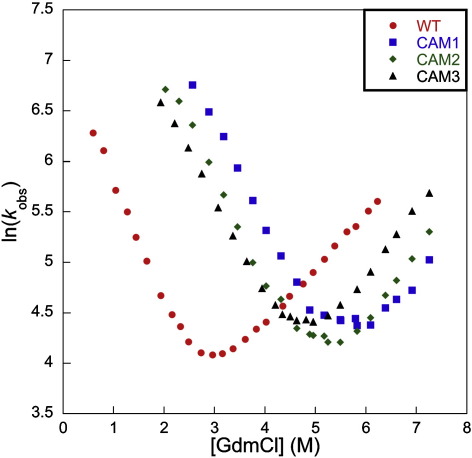
Chevron plots for wild-type LysM and the circular proteins. The circular proteins (CAM1, blue squares; CAM2, green diamonds; CAM3, black triangles) are all 2–3 kcal mol^− 1^ more stable than the wild-type LysM domain (red circles). Units of *k*_obs_ are s^− 1^.

Equilibrium studies showed the LysM domain to be most stable at pH 7, and the folding was completely reversible (data not shown). The kinetics of unfolding are very rapid at 25 °C, and thus all experiments were performed at 10 °C to acquire sufficient data for analysis (Fig. 1). The wild-type equilibrium free energy of unfolding, (3.0 ± 0.1 kcal mol^− 1^), agrees well with that from kinetics (2.9 ± 0.1 kcal mol^− 1^), supporting the conclusion that the folding of LysM is two-state. All mutant protein stabilities were derived from kinetic data, since the low equilibrium *m*-value, (1.1 ± 0.1 kcal mol^− 1^ M^− 1^ in guanidinium chloride, GdmCl), coupled with the low stability of this domain, results in a lack of native baseline in the equilibrium curve of any destabilised mutant.

#### Choice of mutations

The aim of the mutational analysis was to obtain a Φ-value for all core residues and all residues within an element of secondary structure.^27^ Ala → Gly scanning was used to probe the extent of helix formation at all solvent-exposed sites within the α-helices. In total, 48 mutations were performed at 33 different positions within the wild-type polypeptide chain (Table 1). Nine of these mutations resulted in proteins that could be expressed, but had to be isolated from the insoluble fraction after sonication using 6 M GdmCl. In subsequent purification procedures, all of these proteins aggregated, leading to the conclusion that they were unfolded. The contacts lost for each successful mutation are shown in Supplementary Data Table 1.

#### Fitting the kinetic data

The chevron plots for mutants of the wild-type protein are shown in Supplementary Data Fig. 1. None of these mutants exhibit any indication of rollover in the folding arm, and the vast majority of proteins also have completely linear unfolding arms. Any deviation from linearity (e.g., A17G and L37A) is so minor that it cannot be fit reliably to anything but a straight line, and therefore all chevrons were fit to a standard two-state equation. For some of the more destabilised mutants (where ΔΔ*G*_D–N_ > 2 kcal mol^− 1^, e.g., V27A), the denaturation midpoint is shifted to such an extent that there are no data on which to base the folding *m*-value, *m*_*kf*_. To counter this problem all mutants were fit globally with a shared value for *m*_*kf*_, but allowing the unfolding *m*-value, *m*_*ku*_, to vary. Although this global analysis results in minor alterations to the magnitudes of individual Φ-values, the pattern of Φ-values is unchanged (data not shown).

In addition, earlier work by Fersht and co-workers showed that Φ-values calculated at 2 M denaturant are associated with less error than those calculated at 0 M. This is due to the shorter extrapolations needed for *k*_f_ and *k*_u_,^28^ which are used to determine the change in free energy upon mutation (ΔΔ*G*_D–N_). At 2 M denaturant, the standard errors in Φ are low (± 0.05) down to a ΔΔ*G*_D–N_ of ≈ 0.5–0.6 kcal mol^− 1^. Therefore, all Φ-values for the LysM mutants are calculated from rate constants determined at 2 M GdmCl. The results of the kinetic analysis are shown in Table 1.

LysM mutant proteins display a wide range of Φ-values. To reduce the impact of experimental errors, Φ-values are classed as low if they are 0.0–0.2, moderate if they are 0.3–0.5, and high if the Φ-values are 0.6 or above.

#### Mutations within the β-strands

The clearest pattern of Φ-values is seen in β-strand 1 (I3 to V7). All of the Φ-values are low, indicating that there is almost no native-like structure in the transition state. This is true for both core mutations (I3A, I3V, Y5F and V7A) and surface mutations (T4A, T4S and R6A). Strand 2 (D41 to F46) shows moderate Φ-values at its C-terminus (T44A, T44S, L45M, F46L), implying that it is partly structured in the transition state.

#### Mutations within the α-helices

Ala → Gly scanning in helix 1 (L13 to H20) indicates that the helix is almost fully structured in the transition state with high Φ-values for solvent-exposed residues S14 and K18. The buried residues exhibit medium to high Φ-values with the exception of those at the C-terminus (R19 and H20), which have low Φ-values. In helix 2 (I24 to N31), Ala → Gly scanning indicates that the N-terminus is almost fully structured (K25, Φ =  0.7) but that the C-terminus is less well structured (R29, Φ = 0.2). The remaining mutations also support this result, with the highest Φ-value at the N-terminus (I24, Φ = 0.8). Residue S15 exhibits inconsistent behaviour upon mutation (S15A, Φ ≈ 0.0; S15G, Φ = 1.0) and is not included in the global analysis. We have previously observed this behaviour for mutants of another protein, and suggest that it may result from changes in the residual structure of the denatured state.

#### Mutations within the turns and loops

Mutations within the turns and loops span a wide range of Φ-values. The turn between strand 1 and helix 1 is partially structured (R8A, Φ = 0.4; S12A, Φ = 0.6), possibly due to the presence of a salt-bridge between residues R8 and D11. Significant structure is seen also in the turn between helix 1 and helix 2 (G21A, Φ = 0.9; V22A, Φ = 0.5). The long loop between helix 2 and strand 2 seems to have less structure (L37A, Φ = 0.3); however, this loop contains many residues without an associated Φ-value and so this conclusion is more tentative.

### Circular permutation and loop elongation

A circular permutant was created in which the backbone was broken at residue G21 (between the two helices) and a four-residue linker was used to join the original termini in a β-hairpin (see Materials and Methods). The protein was expressed, but was insoluble and prone to aggregation, leading to the conclusion that it was unfolded. Three attempts to extend the inter-helix loop (residue G21 → GSG, GSGSG or GSGSGSG), also resulted in proteins that expressed but were insoluble.

### Studies of the circular protein

#### Choice of circular protein

Three different proteins were created in which the N- and C-termini are linked by a disulphide bond. In protein CAM1, mutations S0C and N49C created a disulphide bond at the domain boundary. For CAM2 the mutations were N(− 1)C and N50C, whilst CAM3 was created with mutations N(− 2)C and N51C. All proteins expressed significantly better than wild type and were much easier to purify (data not shown).

A kinetic analysis of the circular proteins (Fig. 1) identified CAM3 as the best candidate for a protein-engineering study, since it has an unfolding limb that is long enough to obtain a reliable unfolding *m*-value, *m*_*ku*_, for both wild-type and any stabilised mutants. To verify that the increased stability of CAM3 is the result of an intact disulphide bond, its folding was investigated in the presence of 10 mM dithiothreitol (DTT). Under these reducing conditions, the circular protein folded at the same rate as the wild-type domain. Furthermore, there was no indication of any additional phase in the folding or unfolding traces of CAM3 (data not shown).

#### Gross changes upon circularisation

The entropic effect of a disulphide crosslink can be estimated using the method of Pace *et al.*,^29^ which predicts an increase in stability of ≈ 4 kcal mol^− 1^ for each circular variant at 283 K. However, the actual values are slightly lower at 2–3 kcal mol^− 1^ (Fig. 1), possibly because the LysM domain experiences some strain upon formation of the disulphide bond.

CAM3 is stabilised by 2.2 kcal mol^− 1^ upon circularisation of the polypeptide chain, presumably due to the loss in entropy within the denatured state.^29^ The *m*-values for CAM3 and its mutants are consistently lower than those of LysM and its mutants (averages of 1.00 ± 0.05 and 1.17 ± 0.05 kcal mol^− 1^ M^− 1^, respectively). Assuming a similar native state, this indicates that the denatured state of the circular protein is more compact, as expected. Interestingly, the average unfolding *m*-value, *m*_*ku*_, increases slightly from 0.59 ± 0.08 M^− 1^ to 0.70 ± 0.09 M^− 1^. Although these values are within error, the CAM3 unfolding *m*-value is greater in almost all cases (34 out of 38) when we compare the same mutation in LysM and CAM3 (Supplementary Data Fig. 2). We infer that the transition state of the circular protein may be less structured than that of wild-type LysM.

#### Φ-Value analysis of CAM3

To investigate whether the folding transition state of the circular protein, CAM3, is structurally different from that of LysM, 37 mutations were made at 30 positions within the polypeptide chain (Table 2). The chevrons for mutants of the circular protein are shown in Supplementary Data Fig. 3. As with LysM, none of the chevrons exhibit any indication of rollover in the folding arm, nor significant curvature in the unfolding arm. The folding rates for the circular proteins are approximately an order of magnitude faster than those of the linear proteins, and therefore highly destabilised mutants exhibit a very short refolding arm due to the physical limits of the stopped-flow instruments. As before, this problem was overcome by fitting all of the chevrons with a shared value for *m*_*kf*_, but allowing *m*_*ku*_ to vary. The pattern of Φ-values for CAM3 is essentially the same as that of linear LysM. This surprising result is considered in detail in the Discussion.

### Temperature-dependence studies of LysM and CAM3

**Fig. 2:**
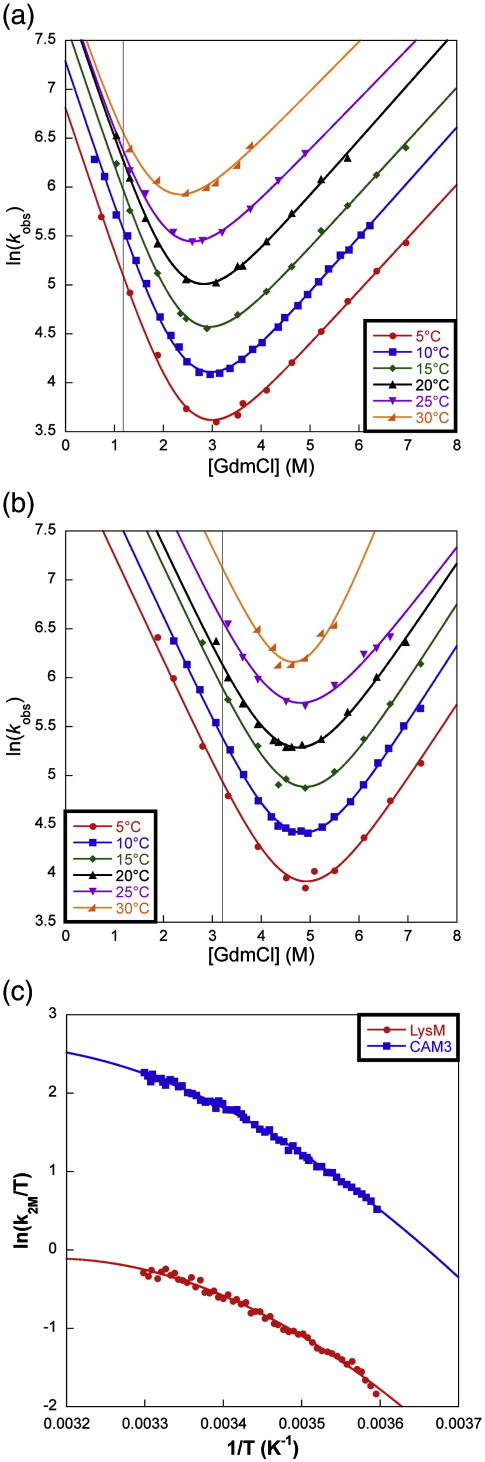
Temperature-dependence studies of LysM and CAM3. a, Chevron plots for the wild-type protein at temperatures between 5 °C (red circles) and 30 °C (orange lower-right triangle). b, Chevron plots for CAM3 at temperatures between 5 °C (red circles) and 30 °C (orange lower-right triangle). All chevrons have been fit to the standard two-state equation, using a fixed value for *m*_*kf*_ (LysM, 1.504 M^− 1^; CAM3, 1.076 M^− 1^). The vertical lines in plots a and b represent the denaturant concentrations at which a more detailed analysis was performed. Units of *k*_obs_ are s^− 1^. c, Eyring plot of the temperature-dependence of the folding rate constants for LysM (red circles) and CAM3 (blue squares) at 2 M GdmCl. The data were determined by extrapolation from the denaturant concentrations at which they were recorded in order to illustrate the refolding rate constants under the conditions chosen for the Φ-value analyses.

For both proteins, unfolding and refolding rates were determined as a function of GdmCl concentration at five different temperatures between 5 °C and 30 °C (Fig. 2). A suitable concentration of denaturant was then chosen at which to perform a more detailed analysis of the temperature-dependence of the folding rate constant. This concentration had to be a compromise between the limits of the stopped-flow instrument, and the requirement to measure folding rates in the linear region of the chevron plot. For LysM, this concentration was chosen to be 1.1 M GdmCl, for CAM3 it was 3.2 M GdmCl (Fig. 2).

The folding rate constants were determined at half-degree intervals between 5 °C and 30 °C at the chosen concentration of denaturant. The rate constants were then extrapolated to 2 M GdmCl to match the conditions under which the Φ-value analyses were performed. An Eyring plot for these data is shown in Fig. 2c. Following the analysis by Chen and co-workers,^30^ the data were fit to:

$$\ln\left(\frac{k_{f}}{T}\right)=\frac{-\Delta C_{p}^{‡}+\Delta S^{‡}(T_{0})}{R}-\ln\frac{h}{k_{B}}+\left(\frac{T_{0}}{T}\right)\left(\frac{\Delta C_{p}^{‡}}{R}-\frac{\Delta H^{‡}(T_{0})}{RT_{0}}\right)+\ln\left(\frac{T_{0}}{T}\right)\left(\frac{-\Delta C_{p}^{‡}}{R}\right)$$

where Δ*C*_*p*_^‡^ is the heat capacity change between the denatured and transition states, Δ*S*^‡^ is the change in entropy between the denatured and transition states, and Δ*H*^‡^ is the change in enthalpy between the denatured and transition states. The reference temperature, *T*_0_, was set at either 10 °C (283K) or 25 °C (298 K) as appropriate. The activation parameters for refolding of LysM and CAM3 are shown in Table 3.

## Discussion

A protein is most likely to exhibit two-state kinetics if it is small (less than 110 residues in length) and has a low level of stability.^1^ The classical example of a two-state folder is CI2,^31^ which comprises just 65 structured residues and has a stability of 7.0 kcal mol^− 1^ under physiological conditions. The lysin motif (LysM) domain is smaller still and has a stability of about 3 kcal mol^− 1^; thus it is not surprising that LysM folds in a two-state manner. However, the interesting question about this domain is the mechanism by which it folds, since the common two-strand-helix nucleation motif is made entropically unfavourable by its β_1_α_1_α_2_β_2_ topology.^5,20^ In addition, the structural symmetry could lead to energetically degenerate transition state ensembles and hence malleability within the folding pathway.^3^ To address these points, an extensive mutational analysis was performed on the wild-type domain.

### The folding transition state of a LysM domain

**Fig. 3:**
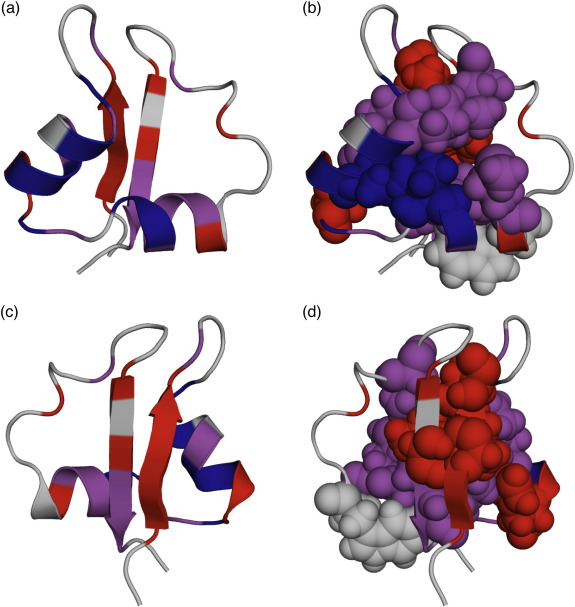
The transition state of LysM. Low Φ-values (0.0–0.2) are shown in red; moderate Φ-values (0.3–0.5) are shown in magenta and high Φ-values are blue. (a) Helix-side view and (c) sheet-side view of LysM showing the Φ-values mapped onto a cartoon representation of the native state structure. (b) Helix-side view and (d) sheet-side view of LysM showing the conserved hydrophobic core residues as filled spheres that are coloured according to their Φ-value. W30 has no Φ-value determined and is shown in grey. These figures were produced with the PyMol Molecular Graphics System [http://pymol.sourceforge.net/].

When mapped onto the native structure of the LysM domain, the Φ-values reveal a highly polarised transition-state structure, with significant formation of the helices but no β-sheet structure (Fig. 3). The highest Φ-values indicate that the critical contacts are between the N-terminus of helix 2 and the central residues of helix 1 (Fig. 3b), resulting in a highly structured helix-turn-helix motif. The C-terminus of helix 2 is unstructured, whereas the C-terminus of helix 1 is structured but has no residue on which to pack: this leads to the low Φ-values of R19 and H20 (Fig. 3d). In addition, the C-terminus of strand 2 shows moderate Φ-values and, since strand 1 is entirely unstructured, this indicates the presence of tertiary interactions between strand 2 and helix 2 (Fig. 3c; Supplementary Data Table 1). This pattern of contacts leads to the identification of a two-helix-strand foldon, which has not been observed for any other protein.

### The rule of three

The highly structured helix-turn-helix motif found for LysM has been observed in the transition state of the all-α FF domain.^32^ A detailed Φ-value analysis on this FF domain showed that the end of helix 1 and the beginning of helix 2 reside in a region of fully formed secondary structure, with well-defined tertiary interactions. As with LysM, analysis of the Φ-values suggested that this helix-turn-helix motif has some interaction with an additional element of secondary structure to complete the folding nucleus. In the case of the FF domain, this additional element is a third helix.

These observations of the transition-state structures of LysM and the FF domain support the hypothesis that residues from at least three topological elements are necessary to create a nucleation-competent submotif (foldon).^11^ Interestingly, this rule of three can be extended to include the transition state of cold-shock protein, which comprises two strands and a distal loop;^33^ and the transition state of α-spectrin SH3, which is formed by a two-strand-3_10_-helix motif.^34^

### Contact order predictions

**Fig. 4:**
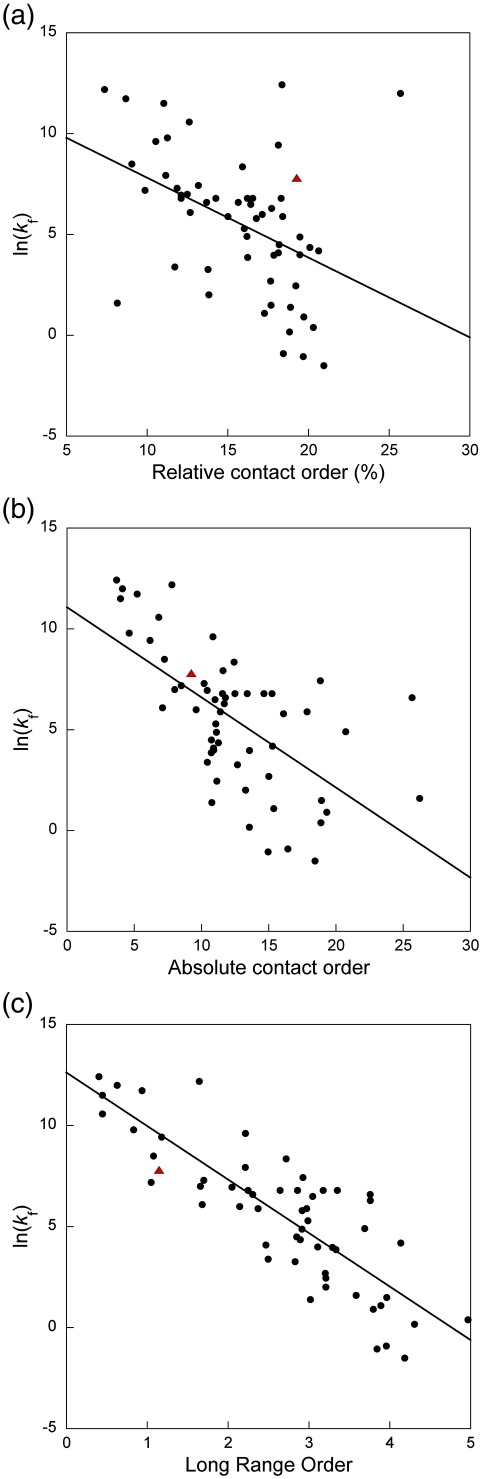
Contact order predictions for LysM. For each plot, the black dots represent proteins from the dataset of Istomim *et al.*^37^, whilst the red triangle represents the LysM domain. a, A plot of RCO^35^ against folding rate. It is clear that LysM folds significantly faster than expected from this prediction method. b, A plot of ACO^36^ against folding rate. c, A plot of LRO^37^ against folding rate. Both ACO and LRO are much better than RCO at predicting the folding rate of the LysM domain. The RCO and ACO were determined using the Baker Laboratory Contact Order Program [http://depts.washington.edu/bakerpg/contact_order/], and the LRO for LysM was determined using the program Insight.

Baker and co-workers analysed the folding of simple, single-domain proteins and revealed a correlation between the rate at which a protein folds, and the topological complexity of its native state.^35^ They introduced the concept of relative contact order (RCO) to quantify this complexity, defined as the average sequence distance between all pairs of contacting residues normalised by the total sequence length. When the RCO of the LysM domain is plotted against the rate at which it folds, it is apparent that the domain folds significantly faster than expected from the contact order plot (Fig. 4a). A subsequent analysis of protein folding rate constants, using a larger dataset, showed that RCO is unable to predict the folding rates of short peptides and large multistate proteins.^36^ The LysM domain, at just 48 structured residues, is similar in size to the small peptides for which the RCO is inaccurate and is much smaller than any protein considered in the initial study.

Based on the wealth of folding data that is now available, two further correlations have been suggested that both show an improvement in the predictions of folding rate constants for all proteins. The first, absolute contact order (ACO), is simply the average sequence distance between all pairs of contacting residues (i.e., not normalised by the total sequence length): larger proteins would be expected to fold more slowly than smaller proteins with a similar topological complexity.^36^ The second correlation, long-range order (LRO), considers only structural contacts that are separated by 12 or more residues.^37^ In contrast to the RCO, both the ACO and LRO structural parameters give significantly improved predictions for the folding rate of the LysM domain (Fig. 4b and c).

Since LysM is one of the smallest domains yet studied by Φ-value analysis, with fewer structured residues than even the engrailed homeodomain,^38^ it is notable that both of these structural parameters are able to estimate its folding rate constant accurately.

### Entropy mutants of LysM

In principle, the LysM protein has four possible, overlapping nucleation motifs: β_1_α_1_α_2_, α_1_α_2_β_2_, α_2_β_2_β_1_ and β_2_β_1_α_1_. We suggest that two of these are disfavoured by the entropic cost of bringing the terminal β-strands together, (α_2_β_2_β_1_ and β_2_β_1_α_1_), which leaves the two contiguous foldon possibilities β_1_α_1_α_2_ and α_1_α_2_β_2_. It is interesting that the dominant foldon is the α_1_α_2_β_2_ motif, since the long α_2_β_2_ loop suggests that this structure should be more entropically costly than the β_1_α_1_α_2_ motif.

We set out to test our hypothesis that the novel two-helix-strand nucleation motif is preferred to the more common two-strand-helix motif due solely to the high entropic cost of formation of the β-sheet. Similar investigations have used circular permutants to test such ideas. These have been termed entropy mutants by Oliveberg and co-workers to reflect the fact that, while the variants produced by circular permutation maintain the spatial contacts between residues, the separation of these residues along the polypeptide chain has altered—thus the chain entropy cost of forming these contacts has changed. Such permutations have been shown to shift the folding nucleus of a protein (e.g., S6^11^ and α-spectrin SH3^10^). We produced the α_2_β_2_β_1_α_1_ circular permutant in an attempt to study an entropy mutant of LysM in which formation of the β-sheet is made more favourable whilst packing of the helices is made more unfavourable. However, this entropy mutant resulted in an unfolded protein. A less severe change in the entropy of the folding nucleus was attempted through elongation of the loop between the two helices, but even an insert of just two residues resulted in a protein that could not be purified. These results indicate that the structure of the loop is critical for correct orientation of the helices, and any disruption to the helix-turn-helix motif produces a domain that is unstable. This hypothesis is supported by the observation that the turn is highly conserved within the LysM superfamily†.^39^ Any LysM domain with an insertion between the two helices is either disulphide cross-linked, or contains multiple proline residues.

It is possible to create an entropy mutant of a domain through circularisation of the polypeptide chain. The transition state of circular CI2 is essentially unchanged from that of the wild-type, although the folding rate is increased sevenfold.^40^ This correlates with the observation that circular permutation of this domain has no effect on the structure of the transition state. In contrast, circularisation of the src SH3 domain changes the transition state from being significantly polarised^41^ to being delocalised. Again, this is consistent with the result that circular permutation of the SH3 domain results in an alteration of the transition state.^10^ Since it is not possible to make the α_2_β_2_β_1_α_1_ circular permutant of LysM, a circularised variant of the domain was achieved by using a disulphide bond to link the N- and C-termini. The topology of this protein, CAM3, is such that none of the four potential nucleation motifs (β_1_α_1_α_2_, α_1_α_2_β_2_, α_2_β_2_β_1_ & β_2_β_1_α_1_) should be significantly disfavoured on the grounds of chain entropy. Indeed, it has been suggested that distant interactions, such as those between the β-strands of LysM, will be energetically stronger than local interactions within a protein domain.^42^ If this were true for LysM, then one might expect circularisation to now favour the two-strand-helix foldons.

### Comparison of LysM and CAM3

**Fig. 5:**
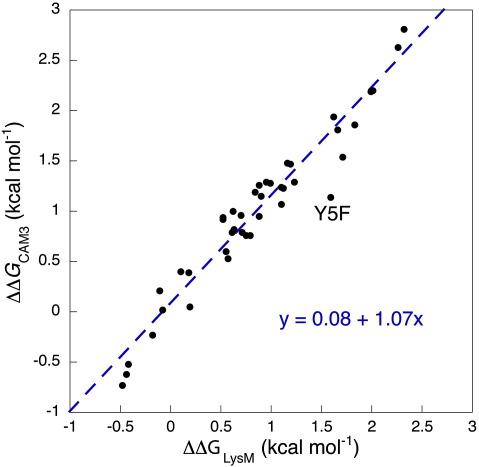
Mutational comparison of LysM and CAM3. ΔΔ*G*_LysM_ refers to the stability change upon mutation within the LysM protein, whilst ΔΔ*G*_CAM3_ is the stability change for the same mutation in CAM3. The extremely strong correlation between the two parameters (*R* = 0.97) is a good indication that the structure of CAM3 is identical with that of LysM. The outlier, Y5F, is associated with an unreliable free-energy change, particularly within the CAM3 variant, since all of its kinetic measurements are based on very fast rates with low amplitudes (Supplementary Data Figs. 1 and 3).

The stability change of the LysM protein upon mutation, ΔΔ*G*_LysM_, is in excellent agreement with that of the equivalent mutation in CAM3 (ΔΔ*G*_CAM3_, Fig. 5; slope = 1.07, *R* = 0.97). This is a strong indication that the structure of the LysM domain is unchanged upon circularisation, and justifies the use of the NMR-derived LysM structure as a pseudo-native state for CAM3. The smaller *m*-value for the circular variant, (1.0 compared to 1.2 kcal mol^− 1^ M^− 1^), thus reflects a more compact CAM3 denatured state, as expected. Neither protein shows any indication of rollover, or any large-scale change in *m*-value upon mutation, leading to the conclusion that both proteins fold through a robust folding pathway with low transition state plasticity.

**Fig. 6:**
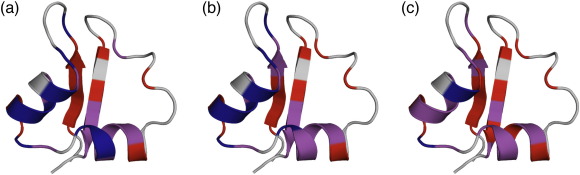
Comparison of the transition states of LysM (‡_LysM_) and CAM3 (‡_CAM3_). In all cases, low Φ-values are mapped onto the native structure in red, moderate Φ-values are shown in magenta, and high Φ-values are blue. a, Representation of ‡_LysM_ where the Φ-value class boundaries are: low, 0.0–0.2; moderate 0.3–0.5; and high, ≥ 0.6. b, Representation of ‡_CAM3_ where the Φ-value class boundaries are: low, 0.0–0.1; moderate 0.2–0.3; and high, ≥ 0.4. The lower boundaries were chosen to facilitate a comparison between the two patterns of Φ-values, since the Φ-values in CAM3 are, on average, 40% lower than those for the equivalent residues in LysM. The pattern of Φ-values in CAM3 and LysM are the same. c, Representation of ‡_CAM3_ coloured according to the LysM Φ-value class boundaries. These figures were produced with the PyMol Molecular Graphics System [http://pymol.sourceforge.net/].

**Fig. 7:**
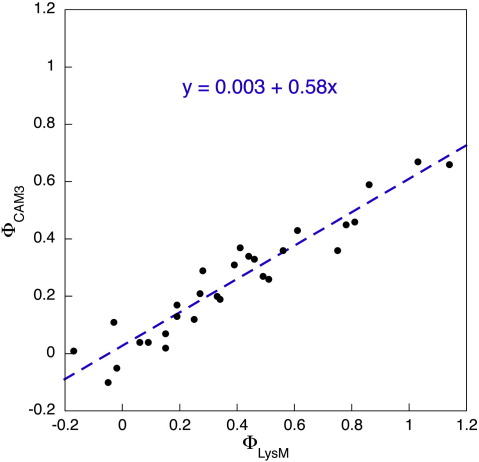
Φ-Value comparison between LysM and CAM3. Φ_LysM_ refers to the Φ-value in the LysM protein, whilst Φ_CAM3_ is the Φ-value associated with the same mutation in CAM3. All Φ-values associated with a ΔΔ*G*_D–N_ < 0.6 kcal mol^− 1^ are excluded from the plot, as is Y5F (see the legend to Fig. 5).

Remarkably, the pattern of Φ-values for the circular protein is essentially identical with that of the linear protein, indicating that the transition state of CAM3 (‡_CAM3_) is qualitatively the same as that seen for the wild-type protein (‡_LysM_; Figs. 6 and 7). In both cases, the critical contacts appear to be between the N-terminus of helix 2 and the central residues of helix 1. Similarly, both proteins show strong evidence that strand 2 is packing onto helix 2 to complete the same two-helix-strand nucleation motif. The resistance of the transition state to both entropy mutations and point mutations leads to the conclusion that the LysM domain comprises only one foldon, and is a rare example of an ideal two-state folder. This unexpected result supports the hypothesis that the energy landscape is dominated by enthalpic rather than entropic considerations, and explains why there is no degeneracy in the folding pathway. Interestingly, since we see no switch to a two-strand-helix foldon, this result contradicts a previous suggestion that long-range interactions in proteins will be more enthalpically favourable than short-range interactions.^42^

A direct comparison of the magnitudes of the Φ-values shows an extremely high correlation (Fig. 7, *R* = 0.95), where Φ_CAM3_ ≈ 0.6. Φ_LysM_. Since the equilibrium stability change upon mutation, ΔΔ*G*_D–N_, is equivalent for the two proteins (Fig. 5), this indicates that the transition state of CAM3 is less sensitive to mutation than that of the wild-type, linear protein. In combination with the observation that the average unfolding *m*-value, *m*_*ku*_, is larger in the circular protein (0.70 *versus* 0.59 M^− 1^ for LysM), this is strong evidence that the transition state of CAM3 is less structured than that of LysM.

### Temperature-dependence studies of LysM and CAM3

To investigate the relative contributions of enthalpy and entropy to the folding of LysM and CAM3, we performed a temperature-dependence study on the folding rate constants of the two proteins (an Eyring analysis^30^; Table 3). Note that in this standard method of analysis, the pre-exponential factor is set as *h*/*k*_B_, which is known to be an overestimation for protein folding reactions. However, we do not require absolute values of *T*Δ*S*^‡^ and Δ*H*^‡^, as we simply wish to make a comparison between LysM and CAM3.

The entropy cost of folding (*T*Δ*S*^‡^) is greater for the linear protein than that for the circular variant. Indeed, at 10 °C and in 2 M GdmCl the folding of LysM is entropically unfavourable (− 1.1 kcal mol^− 1^), whereas the folding of CAM3 is actually favourable (+ 1.1 kcal mol^− 1^). This result is consistent with the denatured state of LysM being higher in entropy than that of CAM3, so that more chain entropy is lost upon folding to the transition state. The overall change in entropy must be dominated by this chain entropy term, since a calculation based on changes in solvent entropy would predict the opposite result.

In terms of enthalpy, both proteins show an unfavourable change on folding to the transition state (Δ*H*^‡^ is positive). This is because, at the free-energy barrier, the nascent protein-protein interactions are not strong enough to fully compensate for the significant loss of protein-solvent and solvent-solvent interactions that are present in the denatured state.

A release of water molecules from solvent clathrates results in an unfavourable increase in solvent enthalpy,^43^ and since the linear protein releases more solvent molecules upon folding to the transition state, if we consider only the solvent, we would expect the enthalpy of folding of wild-type to be more unfavourable than that of CAM3. However, the enthalpy cost of folding is actually greater for the circular variant (13.9 *versus* 13.0 kcal mol^− 1^ at 10 °C and in 2 M GdmCl). The explanation is that there are more enthalpically favourable protein-protein interactions in the transition state of the wild-type protein (‡_LysM_) when compared to that of CAM3 (‡_LysM_). Thus the transition state of LysM must be more structured than that of CAM3, as indicated by the Φ-values.

This analysis supports the observation that the lower entropy cost of formation of the circular transition state is compensated for by a lower enthalpy of contacts within the transition state.

## Conclusions

An extensive mutational analysis of the LysM domain has revealed a novel two-helix-strand nucleation motif (foldon) that has not been observed previously. Entropy mutants of LysM confirm that this two-helix-strand nucleation motif is dominated by enthalpic considerations, and is not simply a result of the LysM topology. The circular protein uses exactly the same foldon as the linear protein and this, combined with the robustness of the transition state, identifies LysM as a rare example of an ideal two-state folder. Importantly, we demonstrate that the lower enthalpy of contacts within the circular transition state compensates for the lower entropic cost of its formation.

## Materials and Methods

### Cloning and mutagenesis

#### Design of the LysM construct

The LysM clone (kindly provided by Dr M. Bycroft, MRC Centre for Protein Engineering), consists of residues 389–452 of the *E. coli* membrane-bound lytic murein transglycosylase D gene (GenBank accession number AAC73316, PDB code 1E0G)^27^. For ease of purification, the gene was inserted between the unique BamHI and EcoRI restriction sites within a modified pRSETA expression vector (Invitrogen: Carlsbad, California), which encodes an N-terminal His-tag and thrombin cleavage site. Thrombin cleavage leaves an N-terminal Gly-Ser extension to the protein. The residue numbering used here is from the first structured residue: Asp398 = Asp1.

#### Design of the circular permutant

The circular permutant consists of residues 22–48, followed by a flexible linker (GSGG) and then residues 1–21. The gene was synthesised using six partially overlapping, synthetic oligonucleotides (Operon GmbH: Cologne, Germany) in a single template generation step. The gene was amplified by PCR and ligated into the modified pRSETA vector (Invitrogen) for expression.

#### Site-directed mutagenesis including loop elongation

Mutagenesis was performed using a Quikchange kit (Stratagene: La Jolla, CA) and was verified by DNA sequencing. This method was used for both point mutations and insertions of up to six codons.

### Protein purification

*E. coli* strain C41(DE3)^44^ was transformed with the appropriate plasmid and the cells were grown at 37 °C in rich medium (2 × TY) containing ampicillin (100 μg ml^− 1^) to an absorbance at 600 nm of 0.4–0.6. Expression was induced by addition of IPTG (0.1 mM final concentration) and the cells were grown overnight at 25 °C before harvesting by centrifugation. The pellets were resuspended in buffer A (20 mM sodium phosphate (pH 7.4), 1 M NaCl), and sonicated on ice. Following centrifugation, the supernatant was incubated for two hours with Ni-NTA resin (Qiagen: Hamburg, Germany). This resin was then washed thoroughly with buffer A containing 25 mM imidazole to remove any weakly bound proteins, before being equilibrated in buffer B (50 mM Tris-HCl (pH 8.0), 1 M NaCl). Then 50 units of thrombin (Sigma: Gillingham, UK) were added per litre of culture, and the resin was kept at 4 °C for 48 h to allow cleavage of the protein from the resin. Following centrifugation, the supernatant was washed through a chilled size-exclusion column with buffer A to separate monomer from aggregate. The appropriate fractions were dialysed extensively against 3-(*N*-morpholino)propanesulfonic acid (MOPS) buffer (50 mM MOPS, pH 7.0) before being flash-frozen in liquid nitrogen and then stored at − 80 °C. Concentrations were estimated using an extinction coefficient of 6970 M^− 1^ cm^− 1^ determined as described.^45^

### Kinetic measurements

Folding was monitored by changes in fluorescence using a 320 nm cut-off filter and an excitation wavelength of 280 nm. All experiments were performed using an Applied Photophysics (Leatherhead, UK) stopped-flow apparatus maintained at 10.5 ± 0.5 °C unless stated otherwise. For unfolding experiments, one volume of 11 μM protein solution was mixed rapidly with ten volumes of a concentrated GdmCl solution. For refolding, one volume of denatured protein (11 μM) in high-concentration GdmCl (typically 6 M or 8 M) was mixed with ten volumes of low-concentration GdmCl. In all cases, both solutions contained MOPS buffer and were chilled in the machine for 2 min before use. Data collected from at least eight experiments were averaged and traces were fit to a single-exponential function. Due to mixing effects, data collected in the first 2.5 ms were always removed before fitting.

### Calculation of Φ-values

All chevron plots were fit to the following equation using Kaleidagraph (Synergy Software, PA):

$$\ln k_{obs}=\left(k_{f}^{2M}\exp\left(-m_{kf}\left(\left[GdmCl\right]-2\right)\right)+k_{u}^{2M}\exp\left(m_{ku}\left(\left[GdmCl\right]-2\right)\right)\right)\text{,}$$

where *k*_obs_ is the observed rate, *k*_f_^2M^ and *k*_u_^2M^ are the folding and unfolding rate constants at 2 M GdmCl, respectively, and *m*_*k*f_ and *m*_*k*u_ are the gradients of the folding and unfolding arms. The stability of each mutant (Δ*G*_D–N_) was calculated using:

$$\Delta G_{D-N}^{2M}=RT\ln\left(k_{f}^{2M}/k_{u}^{2M}\right)$$

and the Φ-value was derived from:

$$\Phi =\ln\left(k_{f}^{2M,mut}/k_{f}^{2M,wt}\right)/\left(\ln\left(k_{f}^{2M,mut}/k_{u}^{2M,mut}\right)-\ln\left(k_{f}^{2M,wt}/k_{u}^{2M,wt}\right)\right)$$

where mut and wt denote the mutant and wild-type protein respectively. The *m*-value for each mutant was calculated using:

$$m_{D-N}=RT(m_{kf}+m_{ku})$$

## Figures and Tables

**Table 1:** Folding data for LysM mutants

	Mutant	*m*_D–N_ (kcal mol^− 1^ M^− 1^)	*k*_f_^2M^ (s^− 1^)	*k*_u_^2M^ (s^− 1^)	Δ*G*_D–N_^2M^ (kcal mol^− 1^)	ΔΔ*G*_D–N_^2M^ (kcal mol^− 1^)	Φ_2M_
	Wild type	1.2	72	26	0.6	–	–
Strand 1	I3A	1.2	54	410	− 1.1	1.7	0.1
	I3V	1.2	75	57	0.2	*0.4*	n.d.
	T4A	1.2	75	92	− 0.1	0.7	0.0
	T4S	1.2	80	55	0.2	*0.4*	n.d.
	Y5F	1.1	96	580	− 1.0	1.6	− 0.1
	R6A	1.1	60	58	0.0	0.6	0.2
	V7A	1.1	38	350	− 1.3	1.8	0.2
Turn 1	R8A	1.1	46	49	0.0	0.6	0.4
	S12A	1.2	8.4	100	− 1.4	2.0	0.6
Helix 1	L13A	1.2	21	270	− 1.4	2.0	0.3
	S14A	1.2	150	24	1.0	− *0.4*	n.d.
	S14G	1.2	43	21	0.4	*0.2*	n.d.
	S14A → G	–	–	–	–	0.6	1.1
	S15A	1.1	77	97	− 0.1	0.7	− 0.1
	S15G	1.1	22	25	− 0.1	0.6	1.0
	S15A → G	–	–	–	–	− *0.1*	n.d.
	I16V	1.2	28	87	− 0.7	1.2	0.4
	A17G	1.3	7.6	150	− 1.7	2.3	0.6
	K18A	1.2	87	26	0.7	− *0.1*	n.d.
	K18G	1.2	19	40	− 0.4	1.0	0.8
	K18A → G	–	–	–	–	1.1	0.8
	R19A	1.2	75	11	1.1	− 0.5	0.1
	H20A	1.2	75	210	− 0.6	1.2	0.0
Turn 2	G21A	1.2	13	34	− 0.5	1.1	0.9
	V22A	1.2	19	120	− 1.0	1.6	0.5
Helix 2	I24A	1.2	18	36	− 0.4	1.0	0.8
	I24V	1.2	63	16	0.8	− *0.2*	n.d.
	K25A	1.1	120	19	1.0	− *0.4*	n.d.
	K25G	1.2	59	25	0.5	*0.1*	n.d.
	K25A → G	–	–	–	–	0.5	0.7
	D26A	1.1	35	51	− 0.2	0.8	0.5
	V27A	1.3	23	520	− 1.7	2.3	0.3
	M28A	1.2	33	58	− 0.3	0.9	0.5
	R29A	1.1	68	34	0.4	*0.2*	n.d.
	R29G	1.2	57	91	− 0.3	0.8	0.2
	R29A → G	–	–	–	–	0.7	0.2
Loop	T34S	1.2	90	78	0.1	0.5	− 0.2
	L37A	1.1	34	240	− 1.1	1.7	0.3
Strand 2	D41A	1.2	93	160	− 0.3	0.9	− 0.2
	L43M	1.2	48	140	− 0.6	1.2	0.2
	T44A	1.1	34	85	− 0.5	1.1	0.4
	T44S	1.3	39	84	− 0.4	1.1	0.3
	L45M	1.1	47	63	− 0.2	0.8	0.3
	F46L	1.2	55	53	0.0	0.6	0.3

*k*_f_^2M^ and *k*_u_^2M^ are given to two significant figures; *m*_D–N_, Δ*G*_D–N_^2M^, ΔΔ*G*_D–N_^2M^ and Φ_2M_ are all given to one decimal place. For clarity, errors are not shown in the Table. The error in free-energy measurements is ± 0.15 kcal mol^− 1^, the error in *k*_f_^2M^ and *k*_u_^2M^ is ± 10% and the error in Φ is ± 0.1. The errors are likely to be much larger for Y5F, since this particular mutant is associated with very fast folding and unfolding rate constants and low amplitudes, (see the chevron plot in Supplementary Data Fig. 1). Mutations with a low change in stability (< 0.5 kcal mol^− 1^) are in italics and their corresponding Φ-values are not determined (n.d.). Mutations G10A, I16A, P39A, P39G, G40A, L43A, L43V, L45A and L45V all resulted in unfolded proteins and are not included in the Table.

**Table 2:** Folding data for CAM3 mutants

	Mutant	*m*_D–N_ (kcal mol^− 1^ M^− 1^)	*k*_f_^2M^ (s^− 1^)	*k*_u_^2M^ (s^− 1^)	Δ*G*_D–N_^2M^ (kcal mol^− 1^)	ΔΔ*G*_D–N_^2M^ (kcal mol^− 1^)	Φ_2M_
	CAM3	1.0	740	5.3	2.8	–	–
Strand 1	I3A	1.1	670	74	1.2	1.5	0.0
	T4A	1.0	620	24	1.8	1.0	0.1
	Y5F	1.1	1400	74	1.6	1.1	− 0.3
	R6A	1.0	540	11	2.2	0.6	0.3
	V7A	0.9	420	82	0.9	1.9	0.2
Turn 1	R8A	1.0	440	13	2.0	0.8	0.4
	S12A	0.9	140	49	0.6	2.2	0.4
Helix 1	L13A	1.0	350	120	0.6	2.2	0.2
	S14A	1.1	1300	3.1	3.4	− 0.6	0.5
	S14G	1.0	400	5.7	2.4	*0.4*	n.d.
	S14A → G	–	–	–	–	1.0	0.7
	S15A	1.0	850	25	2.0	0.8	− 0.1
	S15G	1.0	280	8.6	2.0	0.8	0.7
	S15A → G	–	–	–	–	*0.0*	n.d.
	I16V	1.0	340	24	1.5	1.3	0.3
	A17G	1.0	140	110	0.2	2.6	0.4
	K18A	1.0	750	7.8	2.6	*0.2*	n.d.
	K18G	1.0	320	23	1.5	1.3	0.4
	K18A → G	–	–	–	–	1.1	0.4
	R19A	1.1	780	1.5	3.5	− 0.7	0.0
	H20A	1.0	850	85	1.3	1.5	− 0.1
Turn 2	G21A	1.0	210	13	1.5	1.2	0.6
	V22A	1.0	250	51	0.9	1.9	0.3
Helix 2	I24A	1.0	260	18	1.5	1.3	0.5
	I24V	1.1	600	2.9	3.0	− *0.2*	n.d.
	K25A	1.1	1000	2.9	3.3	− 0.5	0.4
	K25G	1.0	590	8.6	2.4	*0.4*	n.d.
	K25A → G	–	–	–	–	0.9	0.3
	D26A	1.0	520	14	2.0	0.8	0.3
	V27A	0.9	260	280	0.0	2.8	0.2
	M28A	1.0	430	24	1.6	1.2	0.3
	R29A	1.0	820	6.4	2.7	*0.1*	n.d.
	R29G	1.0	710	43	1.6	1.2	0.0
	R29A → G	–	–	–	–	1.1	0.1
Loop	T34S	1.0	660	25	1.8	0.9	0.1
	L37A	0.9	500	91	1.0	1.8	0.1
Strand 2	D41A	0.9	720	49	1.5	1.3	0.0
	L43M	1.0	530	52	1.3	1.5	0.1
	T44A	0.9	370	24	1.5	1.2	0.3
	T44S	1.0	460	18	1.8	1.0	0.3
	L45M	1.0	570	16	2.0	0.8	0.2
	F46L	1.1	580	11	2.3	0.5	0.3

*k*_f_^2M^ and *k*_u_^2M^ are given to two significant figures; *m*_D–N_, Δ*G*_D–N_^2M^, ΔΔ*G*_D–N_^2M^ and Φ_2M_ are all given to one decimal place. For clarity, errors are not shown in the Table. The error in free-energy measurements is ± 0.15 kcal mol^− 1^, the error in *k*_f_^2M^ and *k*_u_^2M^ is ± 10% and the error in Φ is ± 0.1. The errors are likely to be much larger for Y5F, since this particular mutant is associated with very fast folding and unfolding rate constants and low amplitudes, (see the chevron plots in Supplementary Data Fig. 3). Mutations with a low change in stability (< 0.5 kcal mol^− 1^) are in italics and their corresponding Φ-values are not determined (n.d.).

**Table 3:** Activation parameters for the refolding of LysM and CAM3

Protein	[GdmCl] (M)	Temp. (K)	Δ*C*_*p*_^‡^ (kcal mol^− 1^ K^− 1^)	Δ*S*^‡^ (cal mol^− 1^ K^− 1^)	*T*Δ*S*^‡^ (kcal mol^− 1^)	Δ*H*^‡^ (kcal mol^− 1^)	Δ*G*^‡^ (kcal mol^− 1^)
LysM	0	298	− 0.42 ± 0.03	− 19.2 ± 1.2	− 5.7 ± 0.4	6.8 ± 0.4	12.5 ± 0.5
LysM	2	283	− 0.42 ± 0.03	− 3.8 ± 1.2	− 1.1 ± 0.4	13.0 ± 0.3	14.1 ± 0.5
CAM3	0	298	− 0.35 ± 0.04	− 9.5 ± 0.8	− 4.1 ± 0.2	8.8 ± 0.2	11.6 ± 0.3
CAM3	2	283	− 0.35 ± 0.04	4.1 ± 0.8	1.1 ± 0.2	13.9 ± 0.2	12.8 ± 0.3

Note that in this standard method of analysis, the pre-exponential factor is set as *h*/*k*_B_, which is known to be an overestimation for protein folding reactions (see the text).

## Footnotes

† http://pfam.sanger.ac.uk
