# Supplementary material for: Folding of a LysM Domain: Entropy-Enthalpy Compensation in the Transition State of an Ideal Two-state Folder
Source: J Mol Biol. 2008 Jul 11;380(3):557–69. doi: 10.1016/j.jmb.2008.05.020 (PMC2441773; doi:10.1016/j.jmb.2008.05.020)
Supplement: Supplementary material [file mmc1.pdf]

Folding of a LysM domain: entropy-enthalpy compensation in the transition state of an 'ideal' two-state folder

Adrian A. Nickson, Kate E. Stoll[1] and Jane Clarke[†]

University of Cambridge Department of Chemistry, MRC Centre for Protein Engineering, Lensfield Road, Cambridge, CB2 1EW, UK.

[1]Current Address: Department of Biochemistry, Box 357350, University of Washington, Seattle, WA 98195, USA.

[†]Corresponding author; e-mail: jc162@cam.ac.uk; tel: +44 (0) 1223 336426.

Supplementary Materials

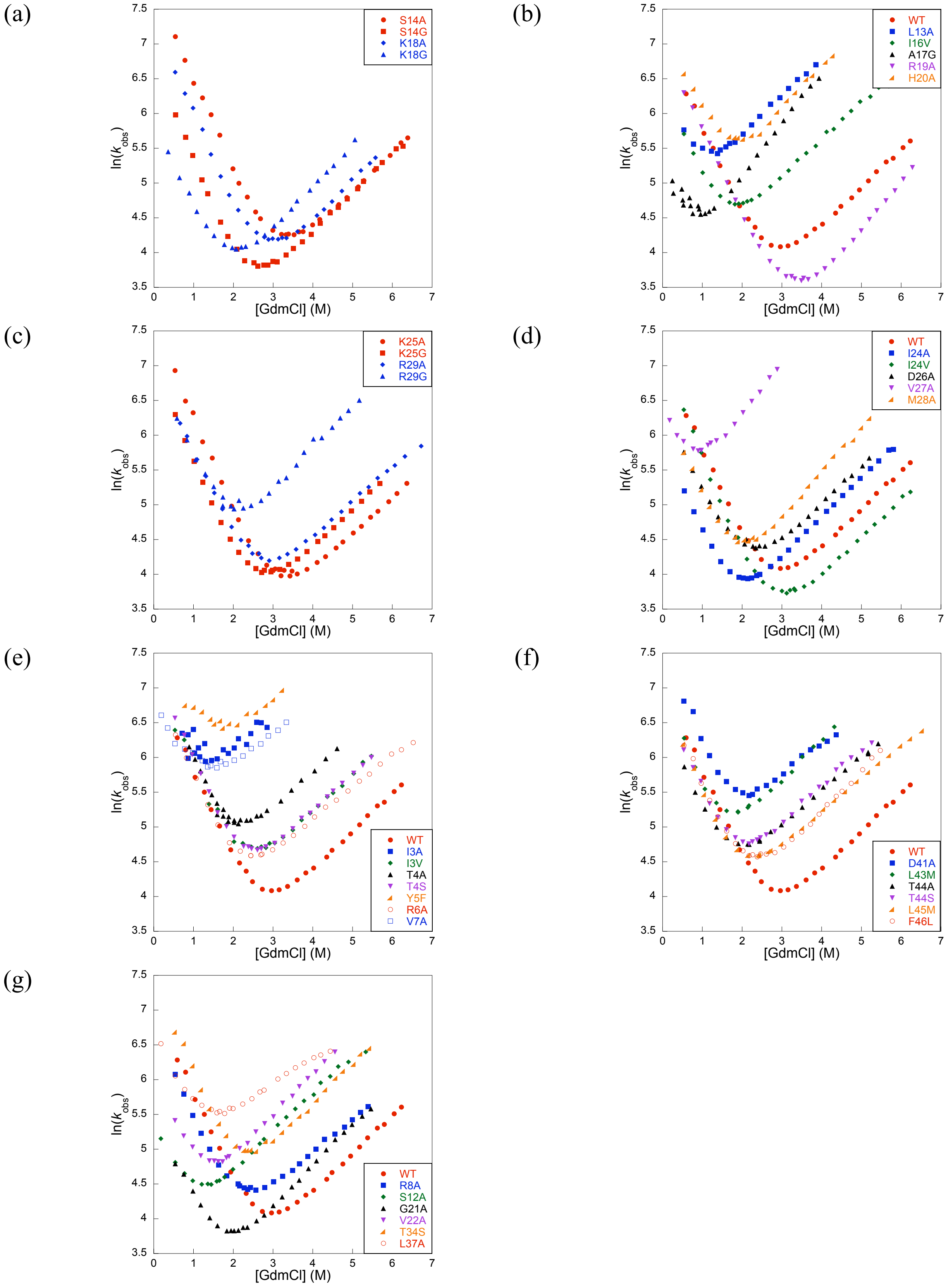

Supplementary Figure 1. Chevron plots for LysM mutants. (a) Helix 1: Ala→Gly scanning mutations. (b) All other mutations in helix 1. (c) Helix 2: Ala→Gly scanning mutations. (d) All other mutations in helix 2. (e) Mutations within β-strand 1. (f) Mutations within β-strand 2. (g) Mutations in the turns and loops between elements of secondary structure.

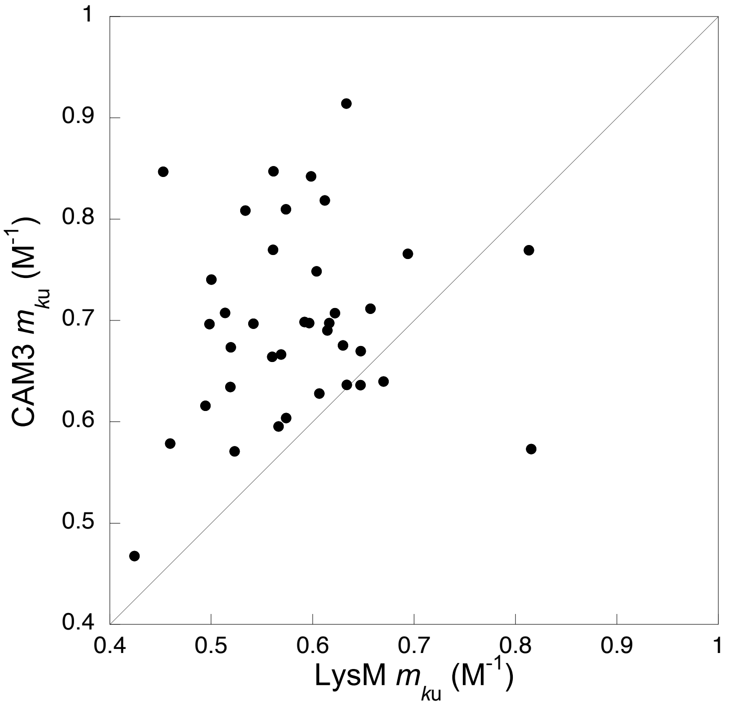

Supplementary Figure 2. Comparison of unfolding *m*-values, m_{k_u}, for LysM and CAM3. The m_{k_u} values from LysM are plotted against the m_{k_u} values of the same mutant in CAM3. The unfolding *m*-values for CAM3 are consistently higher than those for LysM, suggesting that the transition state of CAM3 is less structured than that of LysM.

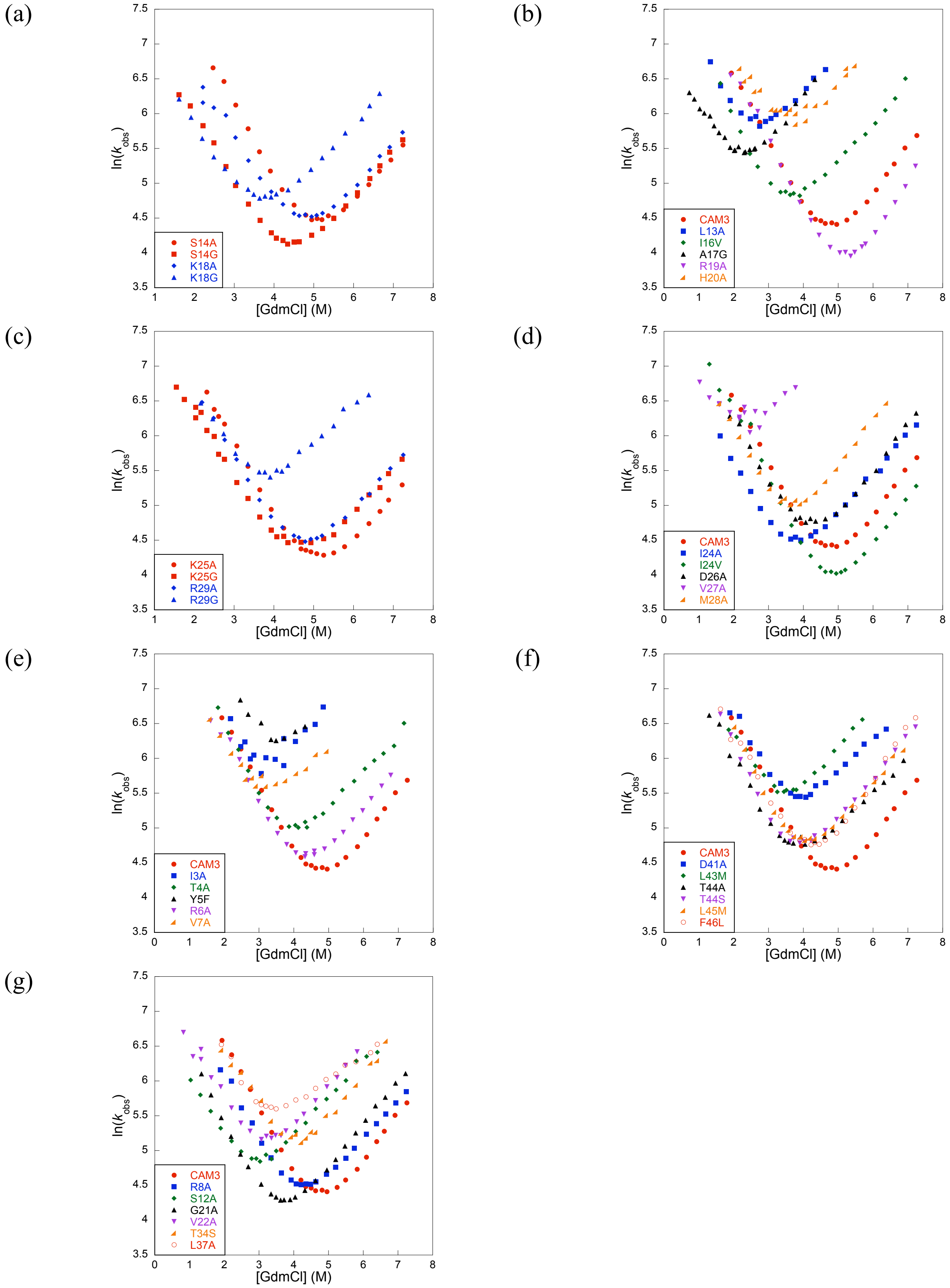

Supplementary Figure 3. Chevron plots for mutants of circular protein CAM3. (a) Helix 1: Ala→Gly scanning mutations. (b) All other mutations in helix 1. (c) Helix 2: Ala→Gly scanning mutations. (d) All other mutations in helix 2. (e) Mutations within β-strand 1. (f) Mutations within β-strand 2. (g) Mutations in the turns and loops between elements of secondary structure.

Supplementary Table 1. Contacts lost upon mutation of LysM.

	Mutant	Residues to which contacts have been lost
Strand 1	I3A	D1 T4 Y5 **H20** V22 **L45** V47 K48
	I3V	D1 H20 V22 L45 V47 K48
	T4A	S2 I3 R6 K42 T44
	T4S	R6 K42
	Y5F	V7 L13 I16 L37 L43
	R6A	T4 D41 **K42**
	V7A	**Y5** R6 D11 L13 I16 L37 L43 D41
Turn 1	R8A	R6 K9 **D11** P39
	S12A	L13 S14 S15
Helix 1	L13A	**Y5** V7 S12 I16 I24 V27 T34 **L37** L43
	S14A	S12 L13 S15 I24
	S14G	S12 L13 S15 A17 I24
	S14A→G	S12 L13 S15 A17 I24
	S15A	D11 S12 I16 K18
	S15G	D11 S12 S14 I16 K18
	S15A→G	S12 S14 I16 K18
	I16V	Y5 V7 D11 S12 L13 S15
	A17G	S14 I16 K18 H20 V22 I24 L45 V47
	K18A	None
	K18G	S15 A17 R19
	K18A→G	S15 A17 R19
	R19A	I16 **H20**
	H20A	**I3 Y5** I16 A17 **R19 V22** I24 **L45** V47
Turn 2	G21A	None
	V22A	I3 I16 A17 H20 I24 V27 L45 V47 K48
Helix 2	I24A	Y5 L13 S14 I16 A17 H20 V22 M28 V27 L45 V47
	I24V	Y5 L13 S14 I16 A17 H20 V22 V27 L45 V47
	K25A	**N23 D26**
	K25G	**N23** I24 **D26** M28
	K25A→G	N23 I24 D26 M28
	D26A	**N23 K25** R29 **W30** V47
	V27A	Y5 L13 V22 I24 D26 M28 W30 N31 T34 L43 T44 L45 V47
	M28A	I24 K25 V27 T34 L37
	R29A	D26 **W30**
	R29G	**D26** M28 **W30**
	R29A→G	D26 M28 W30
Loop	T34S	L13 V27 M28 N31 L37 L43
	L37A	Y5 V7 **L13** M28 T34 N36 D41 **L43**
Strand 2	D41A	N36 Q38 K42
	L43M*	**Y5** V7 L13 V27 N31 T34 N36 L37 T44
	T44A	S2 T4 V27 W30 N31 L45 **F46**
	T44S	S2 V27 W30 N31 L45 F46
	L45M*	I3 **Y5** I16 A17 **H20** V22 I24 V27 L43 V47
	F46L	S2 W30 T44

A contact was counted if it involved two side-chain heavy atoms and fell within a distance cut-off of 6 Å. A minimal loss of interactions between two residues (≤ 5 contacts) is indicated by plain text, a moderate loss (≤ 10 contacts) is indicated by underlined text and significant loss of interactions (> 10 contacts) is indicated in **bold**. *The L→M mutations are non-conservative and so the list of residues shows potential contacts lost, not actual contacts lost.
